# Dr. Lafayette Rinkle

**Published:** 1912-01

**Authors:** 


Dr. Lafayette Rinkle, University of Michigan and Long Island College Hospital, 1874, died at Boonville recently.

